# Maternal feeding practices and toddlers’ fruit and vegetable consumption: results from the DIT-Coombe Hospital birth cohort in Ireland

**DOI:** 10.1186/s12937-021-00743-z

**Published:** 2021-10-19

**Authors:** Xiyao Liu, Qianling Zhou, Keara Clarke, Katherine M. Younger, Meijing An, Zhouyinuo Li, Yang Tan, John M. Kearney

**Affiliations:** 1 Department of Maternal and Child Health, School of Public Health, Peking University, Beijing, China; 2 School of Biological Sciences, Technological University Dublin, Dublin, Ireland; 3 Emma Willard School, Troy, New York, NY USA; 4 Shijiazhuang Foreign Language School, Shijiazhuang, Hebei China

**Keywords:** Toddlers, Feeding, Fruit and vegetable intake, Contribution to total dietary intake, The DIT-Coombe Hospital birth cohort

## Abstract

**Background:**

Health benefits of fruit and vegetable have been well recognized. However, insufficient consumption of fruit and vegetable is prevalent among toddlers, and has become a global public health issue. Maternal feeding practices are potential factors influencing toddlers’ dietary intake, including fruit and vegetable intake. This study was conducted to explore the influence of maternal feeding practices on toddlers’ fruit and vegetable consumption in Ireland.

**Methods:**

A follow-up to the DIT-Coombe Hospital birth cohort was conducted. Mothers in the original cohort were invited to participate in the present follow-up study by phone. A questionnaire assessing maternal feeding behavior and the child’s 3-day food diary was sent to mothers who agreed to take part in the present study by post, together with a self-addressed stamped envelope.

**Results:**

There were 193 mother-children pairs included in the analysis, and the mean age of children was 2.4 (SD 0.7) years old. Toddlers’ mean daily intakes of vegetable and fruit were 67.57 (SD 45.95) g and 213.35 (SD 170.78) g, respectively. Logistic regression analyses showed that maternal practice of breastfeeding for more than 4 weeks was positively associated with fruit (*OR* = 2.93, 95% *CI*: 1.29–6.64) and vegetable (*OR* = 1.95, 95% *CI*: 1.00–3.81) intake or the contribution of fruit (*OR* = 2.62, 95% *CI*: 1.19–5.80) and vegetable (*OR* = 2.02, 95% *CI*: 1.02–3.99) to the total diet. Letting the child eat with other family members was associated with high vegetable intake (OR = 5.45, 95%CI: 1.69–17.61) and high contribution of vegetable to total diet (OR = 3.78, 95% CI: 1.04–13.82). Not being too worried about the child’s refusal to eat was positively associated with toddlers’ vegetable intake (OR = 2.10, 95%CI: 1.09–4.05).

**Conclusions:**

To increase children’s fruit and vegetable intake, and develop good eating habits, parents should eat with their toddlers, be patient and not put much pressure on their children in the context of meal feeding.

**Supplementary Information:**

The online version contains supplementary material available at 10.1186/s12937-021-00743-z.

## Introduction

Toddlerhood is a crucial time for the development of eating patterns. A healthy diet in this period has an impact on children’s health at school-age and adulthood [1, 2]. Fruit and vegetable consumption is essential for a high-quality diet and has many health benefits [3]. Toddlers’ high fruit and vegetable consumption has been found to be associated with a lower BMI and lower risk of obesity [4], and is a protective factor for a variety of chronic diseases (including cardiovascular diseases, diabetes, etc.) [5–7]. What’s more, dietary habits in childhood are likely to track into adulthood [8]. In early childhood, high fruit and vegetable consumption can promote health and reduce the risk of cardiovascular diseases in adulthood [9]. In addition, there was a positive association between diet (e.g. fruit intake) in toddlerhood and cognitive performance at the age of ten [10].

The National Pre-School Nutrition Survey (NPNS), a nationally representative survey in Ireland was conducted between October 2010 and September 2011, to investigate dietary intake of children aged 1–4 years [11, 12]. The NPNS demonstrated that 1) the daily intakes of fruit and vegetable were 190 g and 58 g, respectively; 2) 10% of children didn’t consume vegetable during the four survey days and 12% had very low fruit and vegetable intake (< 10% in the weight of the total dietary intake); and 3) vegetable only accounted for 5% of the total weight of dietary intake. A study in the UK reported that the daily intakes of fruit and vegetable (including all juice) among young children aged 1.5–5 years were 161 g and 66 g, respectively. Inadequate intake and diversity was reported by about half of the children [13]. Similarly, studies in North Carolina, the US (children aged 2–5 years) [14] and the Netherlands [15] (children aged 10–48 months) reported that a large proportion of children did not consume the recommended amount of fruit and vegetable. Therefore, it is necessary to explore the influential factors of toddlers’ fruit and vegetable intake, in order to tailor effective strategies and interventions to promote fruit and vegetable consumption in early childhood.

Parents play a crucial role in shaping children’s early experience with foods [16, 17]. Mothers are the major caregivers of young children, and maternal feeding practices may play the most essential role in toddlers’ dietary intake and weight status [18]. Previous studies found that breastfeeding could promote children’s fruit and vegetable consumption and reduce the intake of unhealthy foods [19, 20]. A study in the US demonstrated that non-directive practices (e.g. education, fewer discipline practices, etc.) of parents were associated with children’s high fruit and vegetable intake [4]. Eating at the table and with the TV off could increase the intake of fruit and vegetable [21]. However, some traditional feeding practices, such as using coercion to promote children’s intake, might be counterproductive and reduce children’s healthy food intake [17]. Therefore, maternal feeding practices play an important role in promoting children’s fruit and vegetable intake, so as to promote healthy nutrition and reduce the risks of obesity. However, to our knowledge, there was a lack of study on the association between maternal feeding practices and toddlers’ dietary intake in Ireland. Besides, relevant studies focused on pre-school and school-age children, but not toddlers.

This study aimed at exploring the relationships between maternal feeding practices and toddlers’ fruit and vegetable intake and their contribution to total dietary intake in the Republic of Ireland. The results of this study might be useful for the improvement of toddlers’ diet quality and health status.

## Materials and methods

### Study design and participants

The DIT-Coombe Hospital study was a birth cohort conducted from June 2004 to October 2006, involving 520 mothers who gave birth in the Coombe Women and Infants University Hospital in Dublin, to explore the prevalence and determinants of breastfeeding and weaning practices in Ireland [22, 23]. This study was a follow-up to the DIT-Coombe Hospital study [22], which was conducted between September 2007 and January 2008. Mothers in the birth cohort were invited to participate in this follow-up study via telephone calls. A questionnaire and a 3-day food diary (including filling instruction and food atlas) were sent to mothers who agreed to participate in this study (*n* = 444) by post, together with a self-addressed stamped envelope. Mothers were required to complete the questionnaire and food diary, and returned them by post. The questionnaire assessed maternal feeding behaviors, and the 3-day food diary documented children’s food intakes.

### Measures

#### Maternal and Children’s characteristics

Maternal and children’s general characteristics included maternal age at childbirth, marital status, education level, occupation, mothers’ country of birth, accommodation, type of delivery, child’s age, and weight at the time of the follow-up study. Information about child’s age and weight was obtained from the present follow-up survey, while other information was collected from the original birth cohort.

#### Maternal feeding practices

Maternal feeding practices were assessed by several questions, which were set in reference to Johnson’s study [24]. For breastfeeding, participants were asked whether or not they had breastfed their child, the duration of breastfeeding for mothers who had breastfed, and the age of infant when solids were introduced. Mothers were further asked to indicate (mark ‘Yes’ or ‘No’) their behaviors if their children refused to eat, including taking it away, coaxing her/him to eat, punishing her/him for not eating, and not worrying too much about it. Moreover, mothers were asked to choose the frequency from ‘never’ to ‘almost all the time’ to the question “If your child is slow to eat or finish a meal, do you try to distract her/him during eating (e.g. watching TV, *etc.*)?” In addition, the question “Does the child usually eat with family members?” was asked. The questionnaire was pilot tested among ten Irish mothers and confirmed good readability and acceptability.

#### Dietary assessment

A standardized 3-day food diary was used to collect food and beverage intakes of the toddlers. The method of 3-day food diary was considered as one of the most accurate methods to reflect true energy intake of young children [25]. Food diary tables together with a filling instruction and a colored-printed food atlas were sent to the participants. The food atlas included photographs of a variety of toddlers’ foods in different portion sizes, which can be converted to the corresponding portion weight. Mothers were required to fill in all foods and beverages consumed by the children for 3 days within a week, including two weekdays and one weekend. At the time of each meal, mothers filled in the type of each food consumed by the child, the preparation method (e.g. fried, ready-made, etc.), brands of the foods, special qualities (e.g. full fat, sugar-free, etc.), and weight consumed. Mothers were required to estimate the food weight consumed using the food atlas and food packaging labels. The occasion and location of each food consumed were also required to be documented. Dietary data was entered into the software WISP© (Tinuviel Software, Llanfechell, Anglesey, UK) by the researcher. The food composition tables used by the WISP© were the sixth edition of McCance and Widdowson’s Food Composition Tables. Vegetables included the edible parts of all kinds of vegetables, excluding potatoes. Potatoes were described separately as a special group. Fruits included the edible parts of all fruits and fruit juice.

#### Outcome variables

Outcome variables included daily vegetable intake, daily fruit intake, the contribution of vegetable to total dietary intake, and the contribution of fruit to total dietary intake. Daily intakes of fruit, vegetable and total diet (g) were the average amount of the 3-day food intake. The contribution of fruit or vegetable to total dietary intake was the proportion (%) of daily fruit or vegetable intake in the total daily dietary intake.

### Statistical analyses

Data analyses were performed using SPSS software, version 23.0 (IBM Corp. Armonk, NY, USA). Continuous variables were presented as Mean (SD), and categorical variables results were presented as percentages. The outcome variables were further categorized into two groups (low v.s. high intake/ contribution). The mean values of daily fruit and vegetable intake and their contribution to the total dietary intake from the National Pre-School Nutrition Survey (NPNS) in Ireland were used as the cut-offs [11, 12]. Therefore, the cut-off values of the daily intakes of fruit and vegetable and their contribution to the total diet were 190 g, 58 g, 15 and 5%, respectively. Univariate analyses were performed to assess the differences in toddlers’ fruit and vegetable intakes and their contribution to total diet across different demographic groups and feeding behavior groups. Logistic regression analyses were performed to explore the relationships between each feeding practice and toddlers’ fruit/ vegetable consumption and their contribution to the total diet. Demographic variables with a significant level of *P* < 0.15 in the univariate analyses were controlled as potential confounders. The use of a univariate *P* value of 0.15 as the threshold for the inclusion of a covariate was in accordance with the recommendation from Bursac et al. [26], in order to avoid missing potentially predictors. The traditional threshold of 0.05 may fail to identify important variables, in particular when our sample size was not very large. In addition, variables that were significantly different between the original cohort and the follow-up study were also controlled in the regression models. A *P* < 0.05 was considered as statistically significant.

## Results

### Participants’ characteristics

A total of 209 mothers returned the questionnaire. After an exclusion of 16 mothers who failed to return the food diaries, 193 mother-child pairs were included in the study. Compared with participants in the original cohort, participants in the follow-up study were more likely to be older, be married, have a higher education level, live on their own property, and have private health insurance (Table S1). Among mothers in the present study, the majority were 25–34 years old, married, of third-level education, and professional/managerial/technical workers. Among 193 children participants, 106 (54.9%) were boys and 87 (45.1%) were girls. The mean age of children was 2.4 (SD 0.7) years old (ranged 1.5–3.4 years old) (Table S1). The mean weight of children was 14.0 (SD 2.7) kg (Table 1). Among mothers included in this study, 42% did not initiate breastfeeding. About 50% of mothers who initiated breastfeeding weaned their children within 3 months and 70% weaned within 6 months. At 6 months, only one mother exclusively breastfed her child (data not shown).

**Table 1:** Participants’ characteristics and differences in fruit and vegetable intakes across characteristics (*n* = 193)

Variables	All participants Mean(SD) or n	Vegetable ^a^				Fruit ^b^			
		Low intake	High intake	*χ*^*2*^*/t*	*P*	Low intake	High intake	*χ*^*2*^*/t*	*P*
		Mean(SD) or n (%)				Mean(SD) or n (%)			
*Child’s age*	2.4(0.7)	2.5(0.7)	2.4(0.7)	0.607	0.544	2.4(0.7)	2.6(0.7)	−1.954	0.052
*Child’s weight (kg)* ^*c*^	14.0(2.7)	14.0(2.4)	14.1(2.9)	−0.547	0.585	13.5(2.4)	14.7(2.8)	−2.981	**0.003**
*Child’s gender*									
Male	106	46(51.1)	60(58.3)	0.989	0.320	58(55.2)	48(54.5)	0.009	0.923
Female	87	44(48.9)	43(41.7)			47(44.8)	40(45.5)		
*Mother’s age at time of childbirth*									
15–24 years old	17	9(10.0)	8(7.8)	5.035	0.081	13(12.4)	4(4.5)	6.711	**0.035**
25–34 years old	123	50(55.6)	73(70.9)			59(56.2)	64(72.7)		
> 34 years old	53	31(34.4)	22(21.4)			33(31.4)	20(22.7)		
*Maternal marital status*									
Married	151	73(81.1)	78(75.7)	0.817	0.366	79(75.2)	72(81.8)	1.217	0.270
Single/divorced/widow	42	17(18.9)	25(24.3)			26(24.8)	16(18.2)		
*Maternal education*									
Primary/Secondary level	54	28(31.1)	26(25.2)	0.936	0.626	34(32.4)	20(22.7)	2.449	0.294
Vocational/training course	56	24(26.7)	32(31.1)			30(28.6)	26(29.5)		
Third level including post graduate	83	38(42.2)	45(43.7)			41(39.0)	42(47.7)		
*Maternal occupation*									
Professional/Managerial/Technical Workers	74	39(43.3)	35(34.0)	2.660	0.447	40(38.1)	34(38.6)	2.853	0.415
Non-Manual	52	24(26.7)	28(27.2)			32(30.5)	20(22.7)		
Skilled Manual/Semi-Skilled	18	6(6.7)	12(11.7)			7(6.7)	11(12.5)		
Students/Unemployed Housewife	49	21(23.3)	28(27.2)			26(24.8)	23(26.1)		
*Maternal birthplace*									
Republic of Ireland	167	79(87.8)	88(85.4)	0.226	0.635	96(91.4)	71(80.7)	4.743	**0.029**
Countries outside Ireland	26	11(12.2)	15(14.6)			9(8.6)	17(19.3)		
*Accommodation*									
Home/Apartment owners	161	78(86.7)	83(80.6)	1.285	0.257	89(84.8)	72(81.8)	0.300	0.584
Non-home owners	32	12(13.3)	20(19.4)			16(15.2)	16(18.2)		
*Health insurance status*									
Public	84	41(45.6)	43(41.7)	0.284	0.867	45(42.9)	39(44.3)	2.756	0.252
Semi-private	71	32(35.6)	39(37.9)			35(33.3)	36(40.9)		
Private	38	17(18.9)	21(20.4)			25(23.8)	13(14.8)		
*Type of delivery*									
Spontaneous Vaginal Delivery	139	67(74.4)	72(69.9)	0.492	0.483	75(71.4)	64(72.7)	0.040	0.841
Caesarean Section	54	23(25.6)	31(30.1)			30(28.6)	24(27.3)		

^a^Vegetable intake was grouped using mean daily intake value in NPNS as cut-off (58 g)

^b^Fruit intake was grouped using mean daily intake value in NPNS as cut-off (190 g)

^c^t’ test

### Fruit and vegetable intakes and their contribution to Total dietary intake

Children’s mean daily intake of vegetable was 67.57 (SD 45.95) g, and the mean daily intake of fruit was 213.35 (SD 170.78) g. The mean daily intake of combined fruit and vegetable was 280.92 (SD 182.71) g; and the mean daily intake of potatoes was 82.08 (SD 53.45) g. The mean daily dietary intake was 1427.38 (SD 334.71) g. The mean contribution of vegetable to total dietary intake was 4.8% (SD 3.2%), and the mean contribution of fruit to total dietary intake was 14.4% (SD 9.6%). The contribution of fruit and vegetable combined to total dietary intake was 19.3% (SD 10.2%) (data not shown).

### Univariate analysis results

Pearson chi-square test and t-test showed that there were no significant relationship between the demographic variables and toddlers’ vegetable intake as well as the contribution of vegetable to total dietary intake (*P* > 0.05). Toddlers’ fruit consumption was significantly associated with children’s weight (*P* = 0.003), maternal birthplace (*P* = 0.029), and mother’s age at the time of childbirth (*P* = 0.035) (Table 1). Children who were heavier, whose mothers were 25–34 years, and whose mothers were from countries outside Ireland were more likely to have higher amount of fruit intake. Older (*P* = 0.003) and heavier (P = 0.003) children were more likely to have higher fruit contribution to total dietary intake (Table 2).

**Table 2:** Participants’ characteristics and differences of fruit and vegetable contribution to total diet across characteristics (n = 193)

Variables	All participants Mean(SD) or n	Vegetable ^a^				Fruit ^b^			
		Low contribution	High contribution	*χ*^*2*^*/t*	*P*	Low contribution	High contribution	*χ*^*2*^*/t*	*P*
		Mean (SD) or n (%)				Mean (SD) or n (%)			
*Child’s age*	2.4(0.7)	2.5(0.7)	2.4(0.7)	0.951	0.343	2.3(0.7)	2.6(0.7)	−3.022	**0.003**
*Child’s weight (kg)*	14.0(2.7)	14.0(2.5)	14.0(3.0)	0.251	0.802	13.5(2.4)	14.8(2.9)	−3.008	**0.003**
*Child’s gender*									
Male	106	62(51.2)	44(61.1)	1.777	0.183	64(57.1)	42(51.9)	0.532	0.466
Female	87	59(48.8)	28(38.9)			48(42.9)	39(48.1)		
*Mother’s age at time of childbirth*									
15–24 years old	17	13(10.7)	4(5.6)	2.908	0.234	12(10.7)	5(6.2)	2.881	0.237
25–34 years old	123	72(59.5)	51(70.8)			66(58.9)	57(70.4)		
> 34 years old	53	36(29.8)	17(23.6)			34(30.4)	19(23.5)		
*Maternal marital status*									
Married	151	94(77.7)	57(79.2)	0.058	0.809	84(75.0)	67(82.7)	1.644	0.200
Single/divorced/widow	42	27(22.3)	15(20.8)			28(25.0)	14(17.3)		
*Maternal education*									
Primary/Secondary level	54	36(29.8)	18(25.0)	0.704	0.703	34(30.4)	20(24.7)	2.319	0.314
Vocational/training course	56	33(27.3)	23(31.9)			35(31.3)	21(25.9)		
Third level including post graduate	83	52(43.0)	31(43.1)			43(38.4)	40(49.4)		
*Maternal occupation*									
Professional/Managerial/Technical Workers	74	48(39.7)	26(36.1)	2.121	0.548	40(35.7)	34(42.0)	4.539	0.209
Non-Manual	52	35(28.9)	17(23.6)			36(32.1)	16(19.8)		
Skilled Manual/Semi-Skilled	18	9(7.4)	9(12.5)			8(7.1)	10(12.3)		
Students/Unemployed/Housewife	49	29(24.0)	20(27.8)			28(25.0)	21(25.9)		
*Maternal birthplace*									
Republic of Ireland	167	104(86.0)	63(87.5)	0.093	0.760	99(88.4)	68(84.0)	0.796	0.372
Countries outside Ireland	26	17(14.0)	9(12.5)			13(11.6)	13(16.0)		
*Accommodation*									
Home/Apartment owners	161	101(83.5)	60(83.3)	0.001	0.980	95(84.8)	66(81.5)	0.379	0.538
Non-home owners	32	20(16.5)	12(16.7)			17(15.2)	15(18.5)		
*Health insurance status*									
Public	84	60(49.6)	24(33.3)	4.864	0.088	50(44.6)	34(42.0)	4.947	0.084
Semi-private	71	40(33.1)	31(43.1)			35(31.3)	36(44.4)		
Private	38	21(17.4)	17(23.6)			27(24.1)	11(13.6)		
*Type of delivery*									
Spontaneous Vaginal Delivery	139	89(73.6)	50(69.4)	0.378	0.539	80(71.4)	59(72.8)	0.046	0.829
Caesarean Section	54	32(26.4)	22(30.6)			32(28.6)	22(27.2)		

^a^Vegetable contribution to total dietary intake was grouped using mean daily intake value in NPNS as cut-off (5.0%)

^b^Fruit intake was grouped using mean daily intake value in NPNS as cut-off (15.0%)

The relationships between maternal feeding practices and toddlers’ fruit and vegetable intakes as well as their contribution to diet were shown in Table 3 and Table 4. Univariate analyses showed that toddlers who were breastfed for over 4 weeks were more likely to consume higher amount of fruit (*P* = 0.005), have higher fruit (*P* = 0.006) and vegetable (*P* = 0.036) contribution to the total diet. Toddlers who usually ate with family members were more likely to have higher amount of vegetable intake (*P* = 0.003) and have higher vegetable contribution (*P* = 0.041) to the total diet. Mother’s behavior of not being too worried when the child refused to eat was associated with high amount of vegetable consumption (*P* = 0.021).

**Table 3:** The relationships between maternal feeding practices and toddlers’ fruit and vegetable intakes, by univariate analyses (n = 193)

Variables	All participants	Vegetable ^a^				Fruit ^b^			
		Low intake	High intake	*χ*^*2*^*/t*	*P*	Low intake	High intake	*χ*^*2*^*/t*	*P*
		n (%)				n (%)			
*Breastfeeding duration*									
< =4 weeks	115	60(66.7)	55(53.4)	3.512	0.061	72(68.6)	43(48.9)	7.722	**0.005**
> 4 weeks	78	30(33.3)	48(46.6)			33(31.4)	45(51.1)		
*Child’s age of weaning onto solid*									
< =16 weeks	119	56(62.2)	63(62.4)	0.000	0.983	68(66.0)	51(58.0)	1.314	0.252
> 16 weeks	72	34(37.8)	38(37.6)			35(34.0)	37(42.0)		
*Does the child usually eat with family members?*									
No, not usually	19	15(16.7)	4(3.9)	8.843	**0.003**	13(12.4)	6(6.8)	1.669	0.196
Yes, most of the time	174	75(83.3)	99(96.1)			92(87.6)	82(93.2)		
*If your child refuses to eat, you tend to*									
Take it away									
No	106	54(62.1)	52(54.7)	1.004	0.316	59(59.0)	47(57.3)	0.052	0.819
Yes	76	33(37.9)	43(45.3)			41(41.0)	35(42.7)		
Coax her/him to eat									
No	56	27(31.4)	29(31.2)	0.001	0.976	36(36.7)	20(24.7)	2.992	0.084
Yes	123	59(68.6)	64(68.8)			62(63.3)	61(75.3)		
Punish her/him for not eating ^c^									
No	175	83(96.5)	92(100.0)	1.499	0.221	94(97.9)	81(98.8)	0.000	1.000
Yes	3	3(3.5)	0(0)			2(2.1)	1(1.2)		
Not worry too much about it									
No	60	36(40.9)	24(25.0)	5.288	**0.021**	36(36.4)	24(28.2)	1.375	0.241
Yes	124	52(59.1)	72(75.0)			63(63.6)	61(71.8)		
*If your child is slow to eat or finish a meal, do you try to distract her/him during eating (*e.g. *playing aeroplanes, watching TV,* etc.*)?*									
Never	50	22(24.4)	28(27.5)	3.767	0.152	23(21.9)	27(31.0)	2.103	0.349
Sometimes	110	48(53.3)	62(60.8)			63(60.0)	47(54.0)		
Quite often/Almost all the time	32	20(22.2)	12(11.8)			19(18.1)	13(14.9)		

^a^Vegetable intake was grouped using mean daily intake value in NPNS as cut-off (58 g)

^b^Fruit intake was grouped using mean daily intake value in NPNS as cut-off (190 g)

^c^Continuity Correction

**Table 4:** The relationships between maternal feeding practices and toddlers’ fruit and vegetable contribution to total diet, by univariate analyses (n = 193)

Variables	All participants	Vegetable ^a^				Fruit ^b^			
		Low contribution	High contribution	*χ*^*2*^*/t*	*P*	Low contribution	High contribution	*χ*^*2*^*/t*	*P*
		n (%)				n (%)			
*Breastfeeding duration*									
< =4 weeks	115	79(65.3)	36(50.0)	4.382	**0.036**	76(67.9)	39(48.1)	7.582	**0.006**
> 4 weeks	78	42(34.7)	36(50.0)			36(32.1)	42(51.9)		
*Child’s age of weaning onto solid*									
< =16 weeks	119	80(66.1)	39(55.7)	2.043	0.153	75(68.2)	44(54.3)	3.816	0.051
> 16 weeks	72	41(33.9)	31(44.3)			35(31.8)	37(45.7)		
*Does the child usually eat with family members?*									
No, not usually	19	16(13.2)	3(4.2)	4.171	**0.041**	9(8.0)	10(12.3)	0.984	0.321
Yes, most of the time	174	105(86.8)	69(95.8)			103(92.0)	71(87.7)		
*If your child refuses to eat, you tend to*									
Take it away									
No	106	68(58.6)	38(57.6)	0.019	0.891	63(57.8)	43(58.9)	0.022	0.882
Yes	76	48(41.4)	28(42.4)			46(42.2)	30(41.1)		
Coax her/him to eat									
No	56	34(30.1)	22(33.3)	0.204	0.651	38(35.5)	18(25.0)	2.213	0.137
Yes	123	79(69.9)	44(66.7)			69(64.5)	54(75.0)		
Punish her/him for not eating ^c^									
No	175	111(97.4)	64(100.0)	0.493	0.483	103(98.1)	72(98.6)	0.000	1.000
Yes	3	3(2.6)	0(0)			2(1.9)	1(1.4)		
Not worry too much about it									
No	60	41(35.0)	19(28.4)	0.866	0.352	38(35.8)	22(28.2)	1.195	0.274
Yes	124	76(65.0)	48(71.6)			68(64.2)	56(71.8)		
*If your child is slow to eat or finish a meal, do you try to distract her/him during eating (*e.g. *playing aeroplanes, watching TV,* etc.*)?*									
Never	50	32(26.7)	18(25.0)	4.664	0.097	26(23.2)	24(30.0)	2.056	0.358
Sometimes	110	63(52.5)	47(65.3)			69(61.6)	41(51.2)		
Quite often/Almost all the time	32	25(20.8)	7(9.7)			17(15.2)	15(18.8)		

^a^Vegetable contribution to total dietary intake was grouped using mean daily intake value in NPNS as cut-off (5.0%)

^b^Fruit intake was grouped using mean daily intake value in NPNS as cut-off (15.0%)

^c^Continuity Correction

### Logistic regression analyses results

Logistic regression analyses results were showed in Table 5. After controlling for potential confounders, maternal practices of breastfeeding for more than 4 weeks (*OR* = 1.95, 95% *CI*: 1.00–3.81), letting the child eat with other family members (*OR* = 5.45, 95% *CI*: 1.69–17.61), and not being too worried about child’s refusal to eat (*OR* = 2.10, 95%*CI*: 1.09–4.05) were positively associated with high amount of vegetable intake. Longer breastfeeding duration was also associated with higher amount of fruit intake (*OR* = 2.93, 95%*CI*: 1.29–6.64) and higher contribution of fruit (*OR* = 2.62, 95% *CI*: 1.19–5.80) and vegetable (*OR* = 2.02, 95% *CI*: 1.02–3.99) to the total dietary intake. Letting the child eat with other family members was positively associated with the contribution of vegetable to total dietary intake (*OR* = 3.78, 95%*CI*: 1.04–13.82).

**Table 5:** The relationships between maternal feeding practices and fruit and vegetable intakes and their contribution to the total dietary intake, by multivariate analyses

Variables	Intake				Contribution to total dietary intake			
	Vegetable		Fruit		Vegetable		Fruit	
	Adjusted *OR* (95% *CI*)	*P*	Adjusted *OR* (95% *CI*)	*P*	Adjusted *OR* (95% *CI*)	*P*	Adjusted *OR* (95% *CI*)	*P*
Breastfeeding duration (> 4 weeks)	**1.95** **(1.00–3.81)**	**0.050**	**2.93** **(1.29–6.64)**	**0.010**	**2.02** **(1.02–3.99)**	**0.043**	**2.62** **(1.19–5.80)**	**0.017**
Child’s age of weaning onto solid (> 16 weeks)	1.15 (0.59–2.21)	0.687	1.15 (0.54–2.47)	0.723	1.81 (0.92–3.58)	0.087	1.79 (0.81–3.99)	0.151
Does the child usually eat with family members? (Yes, most of the time)	**5.45** **(1.69–17.61)**	**0.005**	2.14 (0.66–6.90)	0.205	**3.78** **(1.04–13.82)**	**0.044**	0.68 (0.22–2.09)	0.501
*If your child refuses to eat, you tend to*								
Take it away	1.33 (0.70–2.53)	0.390	0.95 (0.45–1.99)	0.882	0.95 (0.49–1.85)	0.886	0.93 (0.43–2.03)	0.858
Coax her/him to eat	0.96 (0.50–1.86)	0.909	1.77 (0.80–3.90)	0.157	0.84 (0.43–1.66)	0.621	1.71 (0.75–3.90)	0.203
Punish her/him for not eating	NA	NA	0.580 (0.05–7.31)	0.674	NA	NA	0.67 (0.05–8.47)	0.758
Not worry too much about it (Yes)	**2.10** **(1.09–4.05)**	**0.027**	1.48 (0.69–3.18)	0.313	1.30 (0.66–2.58)	0.446	1.04 (0.47–2.26)	0.930
*If your child is slow to eat or finish a meal, do you try to distract her/him during eating (*e.g. *playing aeroplanes, watching TV,* etc.*)?*								
Sometimes	1.00 (0.49–2.04)	0.994	0.54 (0.24–1.23)	0.140	1.23 (0.59–2.57)	0.574	0.66 (0.28–1.55)	0.339
Quite often/Almost all the time	0.47 (0.18–1.20)	0.115	0.39 (0.13–1.16)	0.091	0.45 (0.16–1.31)	0.144	0.93 (0.32–2.74)	0.899

The independent variable was each feeding practices variable, with the adjustment of demographic variables with a significant level of *P* < 0.15 in the univariate analyses (Table 1 and Table 2) and significantly different between the original study and follow-up study (Table S1) (i.e. mother’s age at time of childbirth, maternal marital status, maternal education, accommodation and health insurance status)

## Discussion

This study provided evidence about the relationships between maternal feeding practices and toddlers’ fruit and vegetable intakes. The results showed that breastfeeding for more than 4 weeks, letting the child eat with other family members, and not being too worried about child’s refusal to eat were positively associated with high amount of vegetable consumption. Longer duration of breastfeeding was positively associated with fruit intake and the contribution of fruit and vegetable to total diet. Toddlers who ate with other family members were more likely to have high vegetable contribution to total diet.

Growing children need foods high in nutrient density but moderate in energy density, such as fruit and vegetable [17]. Therefore, toddlers need to consume enough fruit and vegetable. The NPNS [11] studied fruit and vegetable intakes, and their contribution to the total diet among Irish young children aged 1–4 years. In their study, children’s mean daily intake of fruit and vegetable combined was 248 g/d (190 g came from fruit) and contributed to 20% (15% came from fruit) of the total diet. In the UK National Diet and Nutrition Survey [27], the mean fruit and vegetable intake of all participants aged 1.5–3 years was 251 g/d (179 g was from fruit). In the present study, the total intake of fruit and vegetable was higher than that reported by studies in Ireland and the UK, and the contribution of fruit and vegetable to the total dietary intake was similar to the result of the NPNS [11]. In the present study, the toddlers’ daily intake of fruit was much higher than the intake of vegetable. There was little difference in daily fruit and vegetable intakes among the above three studies, which may be due to the differences of the participants’ characteristics. The age of children varied and the regions were different. The variations in results could also be contributed by random errors in dietary assessment methods. The Dietary Guidelines for Americans (DGA) suggests a minimum consumption of 1 to 1.5 cups of fruit and 1 to 1.5 cups of vegetable daily for children aged 2–8 [28]. According to the previous studies and guidelines [29, 30], a cup of vegetable is equal to approximately 76–152 g and a cup of fruit/fruit juice is equal to approximately 140–210 g. According to the present and the previous studies, insufficient intake of vegetable was serious among children. In the present study, toddlers’ intake of potatoes was more than the total intake of other vegetables. Many nutrition surveys and dietary guidelines exclude potatoes from the vegetable group because of their special composition. Besides, although potatoes are rich in a variety of nutrients, unhealthy preparation methods such as frying, may be popular in children’s diet [31]. As a result, children should be encouraged to consume other types of vegetable. At the time of this study, dietary guidelines in Ireland were for population over 5 years [32, 33], and those for toddlers were lacking. Toddlerhood is a crucial period for individual’s growth and development, and the establishment of healthy eating behaviors [1, 2]. Therefore, it is necessary to develop dietary guideline for toddlers.

As is well-known, breastfeeding is beneficial for children. The WHO and UNICEF recommend exclusive breastfeeding during the first 6 months of life. After 6 months, infants should be breastfed for up to 2 years and beyond [34]. A number of studies have been conducted to examine whether breastfeeding could influence children’s dietary intake. Hamulka et al. [19] and Specht et al. [35] found that there were positive associations between breastfeeding and vegetable consumption among children aged 7–12 years and 2–6 years, respectively. Perrine et al. [20] reported that breastfeeding promoted both fruit and vegetable intakes among children at the age of six. Soldateli et al. [3] and Moss et al. [36] showed that a longer duration of breastfeeding was positively associated with higher amount of vegetable consumption and the variety of vegetable consumed in children. In the present study, breastfeeding duration of most participants was much shorter than the recommendation, and only one mother breastfed exclusively for 6 months. Nevertheless, breastfeeding duration was found to be positively associated with fruit and vegetable intakes, and their contribution to total the dietary intake. Our result was in consistent with the literature. It is likely that the flavors of food consumed by the mothers could be transmitted to their children through breast milk. Longer breastfeeding duration makes healthy food more familiar and acceptable by the children [37]. Besides, Forestell and Mennella [38] believed that the initial advantages of breastfeeding must be followed by repeated exposure to fruits and vegetables after solids were introduced. Therefore, the practices of breastfeeding and feeding children with healthy foods should be promoted.

The environment created by parents may be an important factor associated with children’s dietary intake. In the present study, toddlers who ate with other family members had high vegetable intake and high contribution of vegetable to total diet. The results implied that letting the child eat with other family members may promote toddlers’ vegetable intake. Our finding was consistent with the studies of Andaya et al. [39], Mak et al. [21], and Hammons et al. [40]. Andaya et al. [39] found that children who often ate with family members were more likely to consume fruit and vegetable. Mak et al. [21] showed that eating at the table could increase children’s consumption of fruit and vegetable because family meals usually occurred around the table. In the review of Hammons et al. [40], children and adolescents who ate with family members more times per week were more likely to be in normal weight, have healthier dietary patterns, and have a lower risk of eating disorders. Parents are often children’s role models of healthy eating, and children’s fruit and vegetable consumption is associated with parental consumption [41]. Children who eat with their families are more likely to be encouraged to try new foods, and maybe more receptive to fruit and vegetable. Therefore, when children are old enough to try a variety of foods, they should be encouraged to eat with family members to develop good eating habits. During this process, parents should also be careful to prevent adults’ unhealthy eating behavior from affecting their children. Besides, entertainment during meal time also has an impact on children’s diet [42]. In this study, if children were slow to eat or finish a meal, mothers’ using distraction strategy (e.g. TV, smartphone) was not associated with fruit and vegetable consumption. Our result was in contrast to previous studies. Campbell et al. [42] stated that children who ate with the TV off and not moving around were more likely to eat fruit and vegetable. Likewise, Mak et al. [21] reported that eating with TV off was also a promoting factor in children’s vegetable consumption. The differences between our findings and the previous studies might because mothers’ distraction strategy can increase young children’s fruit and vegetable intakes temporarily. To sum up, mothers should create a focused and healthy eating environment, which could help young children develop correct eating habits and increase the intake of healthy foods.

When children refuse to eat, parents may feel worried and anxious, and try to increase their children’s dietary intake. However, excessive prodding or forcing may reduce children’s intake of healthy foods. In our study, mothers’ not being too worried about children’s refusal to eat was positively associated with toddlers’ vegetable intake and vegetable contribution to total diet. There is evidence that a mother who worries excessively about child’s under-eating is more likely to force her child to eat more foods, and mother’s negative emotion could be conveyed to her child or make her child feel stressed. Fries et al. [43] proposed that coercive feeding practices, such as the use of pressure to eat, might lead to children’s food rejection [44]. Kristiansen et al. [45] stated that encouragement can increase children’s vegetable intake, but the tendencies to nagging might be counterproductive. Fisher et al. [46] showed that both parents’ and children’s fruit and vegetable consumption was reduced when eating under great pressure. Galloway et al. [47] indicated that pressure negatively affected children’s affective responses to and consumption of healthy foods. As a result, mothers should be patient and avoid negative emotion when they are feeding their children. Mothers should also pay attention to children’s hunger and satiety signals, and find out the reasons for food refusal and feed correctly when the children refuse to eat. Pressuring children to eat may increase their intake temporarily, but could impact negatively on children’s long-term healthy diet and cause food neophobia [47, 48].

For demographic variables, maternal education and income level have been demonstrated to be associated with children’s fruit and vegetable consumption. Burnier et al. [49] and Cooke et al. [41] stated that mothers’ high education level was related to children’s high vegetable intake. Laitinen et al. [50] also reported that children from families with higher income ate more fruit. However, in the present study, variables related to family income (i.e. accommodation and health insurance status) and education level were not related to children’s fruit and vegetable intakes, but only related to the contribution of vegetable to the total diet. It might be due to the participants in this follow-up study were from the same hospital and thus have similarities in services and education. However, the vegetable contribution was influenced by dietary diversity, which might be related to education and income level.

The strengths of this study include using a standardized 3-day food diary to assess children’s fruit and vegetable consumption, and providing evidence for the relationships between maternal feeding practices and children’s dietary intake. The 3-day food diary is the most accurate method to assess dietary intake, and our dietary data is thus reliable. Besides, this study adopted a longitudinal design, and some causal relationships could thus be inferred.

However, our study has some limitations. First, this study was conducted between 2007 and 2008. There may be some differences with the current situation. Second, the self-reported data might be prone to bias. Third, the sample size of this study was small, and the social disability bias might exist. It was burdensome for mothers to complete a 3-day food diary. The questionnaires were distributed and collected by mail. As a result, although we tried to get as many participants as possible to send back the questionnaires (i.e. re-contacting the participants by phone calls, resending questionnaire to participants whose questionnaire/food diary was lost), there were only 43.5% of mothers returned the food diary. Results of this study could serve as a template for further exploration between maternal feeding practices and dietary intake of young children with a larger sample size.

## Conclusion

Our study revealed that maternal practices of breastfeeding for more than 4 weeks, letting the child eat with other family members, and not being too worried about child’s refusal to eat were positively associated with toddlers’ vegetable intake. Breastfeeding duration was also positively associated with fruit intake and fruit contribution to total dietary intake. The practice of letting the child to eat with other family members was related to high vegetable contribution to total dietary intake. To increase children’s fruit and vegetable intake and develop good eating habits, parents might eat with their toddlers, be patient, and not put much pressure on their children when feeding. Further studies might consider having a larger sample size to verify the findings of our study.

## Supplementary Information

**Additional file 1: Table S1** Socio-demographic characteristics of participants of the DIT-Coombe Cohort study (*n* = 520), respondents (*n* = 193) and non-respondents (*n* = 327) of the present follow-up study.

## Data Availability

The dataset for the present study is available from the corresponding author upon reasonable request.
