# Supplementary material for: Maternal feeding practices and toddlers’ fruit and vegetable consumption: results from the DIT-Coombe Hospital birth cohort in Ireland
Source: Nutr J. 2021 Oct 19;20:84. doi: 10.1186/s12937-021-00743-z (PMC8524861; doi:10.1186/s12937-021-00743-z)
Supplement: Supplementary file 1 — Additional file 1: Table S1 Socio-demographic characteristics of participants of the DIT-Coombe Cohort study (n = 520), respondents (n = 193) and non-respondents (n = 327) of the present follow-up study. [file 12937_2021_743_MOESM1_ESM.docx]

**Table S1** Socio-demographic characteristics of participants of the DIT-Coombe Cohort study (n=520), respondents (n=193) and non-respondents (n=327) of the present follow-up study.

| Variables | Participants of the DIT-Coombe Cohort study (n=520) | Respondents to the present follow-up study (n=193) | Non-respondents to the present follow-up study (n=327) | *χ^2^/t* | *P* |
| --- | --- | --- | --- | --- | --- |
|  | n (%) or Mean (SD) | | |  |  |
| *Mother’s age at time of childbirth* |  |  |  |  |  |
| 15-24 years old | 107 (20.6) | 17(8.8) | 90(27.5) | 14.740 | **0.001** |
| 25-34 years old | 306 (58.8) | 123(63.7) | 183(56.0) |  |  |
| >34 years old | 107 (20.6) | 53(27.5) | 54(16.5) |  |  |
| *Maternal marital status* |  |  |  |  |  |
| Married | 322 (61.9) | 151(78.2) | 171(52.3) | 16.779 | **<0.001** |
| Single/divorced/widow | 198 (38.1) | 42(21.8) | 156(47.7) |  |  |
| *Maternal education* |  |  |  |  |  |
| Primary/Secondary level | 200 (38.5) | 54(28.0) | 146(44.6) | 7.561 | **0.023** |
| Diploma | 143 (27.5) | 56(29.0) | 87(26.6) |  |  |
| Third level | 177 (34.0) | 83(43.0) | 94(28.7) |  |  |
| *Maternal occupation* |  |  |  |  |  |
| Professional/Managerial  /Technical Workers | 161 (31.0) | 74(38.3) | 87(26.6) | 3.853 | 0.278 |
| Non-Manual | 145 (27.9) | 52(26.9) | 93(28.4) |  |  |
| Skilled Manual/Semi-Skilled | 57 (11.0) | 18(9.3) | 39(11.9) |  |  |
| Students/Unemployed  /Housewife | 157 (30.2) | 49(25.4) | 108(33.0) |  |  |
| *Accommodation* |  |  |  |  |  |
| Home/Apartment owner | 361 (69.4) | 161(83.4) | 200(61.2) | 14.060 | **<0.001** |
| Non-home owners | 159 (30.6) | 32(16.6) | 127(38.8) |  |  |
| *Health insurance status* |  |  |  |  |  |
| Public | 277 (53.3) | 84(43.5) | 193(59.0) | 6.079 | **0.048** |
| Semi-private | 169 (32.5) | 71(36.8) | 98(30.0) |  |  |
| Private | 74 (14.2) | 38(19.7) | 36(11.0) |  |  |
| *Maternal birthplace* |  |  |  |  |  |
| Republic of Ireland | 454 (87.3) | 167(86.5) | 287(87.8) | 0.076 | 0.783 |
| Countries outside Ireland | 66 (12.7) | 26(13.5) | 40(12.2) |  |  |
| *Child’s age when the follow-up study was conducted (years)* | 2.4(0.7) | 2.4(0.7) | 2.4(0.7) | -0.458 | 0.647 |
| *Chid’s gender* |  |  |  |  |  |
| Male | 283 (54.4) | 106(54.9) | 177(54.1) | 0.014 | 0.905 |
| Female | 237(45.6) | 87(45.1) | 150(45.9) |  |  |
| The values of *χ^2^/t* and *P* presented the relationship between participants of the DIT-Coombe Cohort study (n=520) and respondents (n=193) of the present study. | | | | | |
